# Gastric metastasis from pancreatic ductal adenocarcinoma: a case report

**DOI:** 10.1093/jscr/rjag233

**Published:** 2026-04-11

**Authors:** Takashi Urano, Atsushi Urakami, Munenori Takaoka, Akihiro Shibuya, Teppei Onishi, Akihisa Akagi, Noriyo Urata, Hirofumi Kawamoto, Takashi Akiyama, Tomoki Yamatsuji

**Affiliations:** Department of General Surgery, Kawasaki Medical School General Medical Center, 2-6-1 Nakasange, Kita-ku, Okayama 700-8505, Japan; Department of General Surgery, Kawasaki Medical School General Medical Center, 2-6-1 Nakasange, Kita-ku, Okayama 700-8505, Japan; Department of General Surgery, Kawasaki Medical School General Medical Center, 2-6-1 Nakasange, Kita-ku, Okayama 700-8505, Japan; Department of General Surgery, Kawasaki Medical School General Medical Center, 2-6-1 Nakasange, Kita-ku, Okayama 700-8505, Japan; Department of General Surgery, Kawasaki Medical School General Medical Center, 2-6-1 Nakasange, Kita-ku, Okayama 700-8505, Japan; Department of General Surgery, Kawasaki Medical School General Medical Center, 2-6-1 Nakasange, Kita-ku, Okayama 700-8505, Japan; Department of General Internal Medicine, Kawasaki Medical School General Medical Center, 2-6-1 Nakasange, Kita-ku, Okayama 700-8505, Japan; Department of General Internal Medicine, Kawasaki Medical School General Medical Center, 2-6-1 Nakasange, Kita-ku, Okayama 700-8505, Japan; Department of Pathology, Kawasaki Medical School General Medical Center, 2-6-1 Nakasange, Kita-ku, Okayama 700-8505, Japan; Department of General Surgery, Kawasaki Medical School General Medical Center, 2-6-1 Nakasange, Kita-ku, Okayama 700-8505, Japan

**Keywords:** gastric metastasis, pancreatic ductal adenocarcinoma, pancreatic cancer, stomach, conversion surgery

## Abstract

Pancreatic ductal adenocarcinoma (PDAC) rarely metastasizes to the gastrointestinal tract. Herein, we report a case of gastric metastasis from PDAC. An 83-year-old woman presented with a mass in the pancreatic tail. CT revealed a 5 cm neoplastic lesion in the pancreatic tail invading the splenic vessels and parenchyma, with lymph node metastasis. Collateral veins developed in the posterior stomach wall nine courses of chemotherapy were administered. After chemotherapy, esophagogastroduodenoscopy revealed an irregularly elevated lesion in the gastric body, which was identified as an adenocarcinoma. These lesions were considered resectable, and surgery, including distal pancreatectomy, splenectomy, lymph node dissection, and partial gastrectomy, was performed. Pathological examination revealed that the stomach wall had an extension of well-differentiated adenocarcinoma, mainly in the submucosa. The immunohistochemical staining pattern was similar to PDAC; therefore, gastric metastasis was diagnosed. The final pathological diagnosis was pT3N1M1 (stomach, LYM), pStage IV.

## Introduction

Metastatic gastric tumors are uncommon. Metastatic lesions in the stomach are found in 0.2%–1.7% of the population in autopsy series [1]. Additionally, pancreatic ductal adenocarcinoma (PDAC) often metastasizes to the liver, peritoneum, lymph nodes, and lungs but rarely metastasizes to the gastrointestinal tract. Gastric metastasis has been reported in only two of 209 autopsies (0.96%) of pancreatic cancer [2]. Herein, we report a case of gastric metastasis from PDAC in the pancreatic tail that was surgically resected. The patient survived for more than 2 years.

## Case report

**Figure 1:**
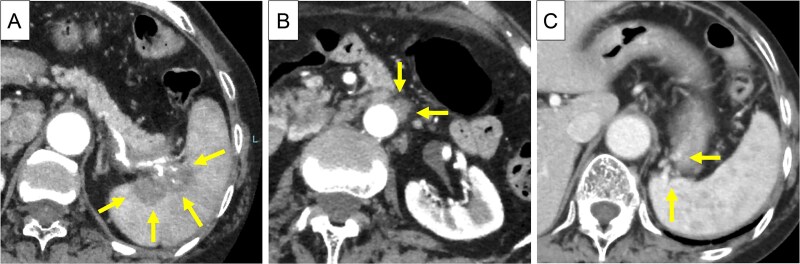
CE-CT before chemotherapy. (A) A 5 cm-sized neoplastic lesion in the pancreatic tail, with invasion into the splenic artery and splenic parenchyma (arrows). (B) the Para-aortic lymph nodes were enlarged (arrows). (C) Due to obstruction of the splenic vein at the hilum of the spleen, collateral veins had developed in the posterior wall of the stomach (arrows).

An 83-year-old Japanese woman with a history of cerebral hemorrhage was referred to our hospital because of a mass identified in the pancreatic tail while undergoing a computed tomography (CT) scan at a local hospital due to chest tightness. She did not have abdominal pain, and her CA19–9 level was 57.3 U/ml. Contrast-enhanced (CE)-CT showed a 5 cm-sized neoplastic lesion in the pancreatic tail, with invasion into the splenic vessels and parenchyma (Fig. 1A) and para-aortic lymph node swelling (Fig. 1B). Collateral veins had developed within the wall of the upper gastric body (Fig. 1C).

**Figure 2:**
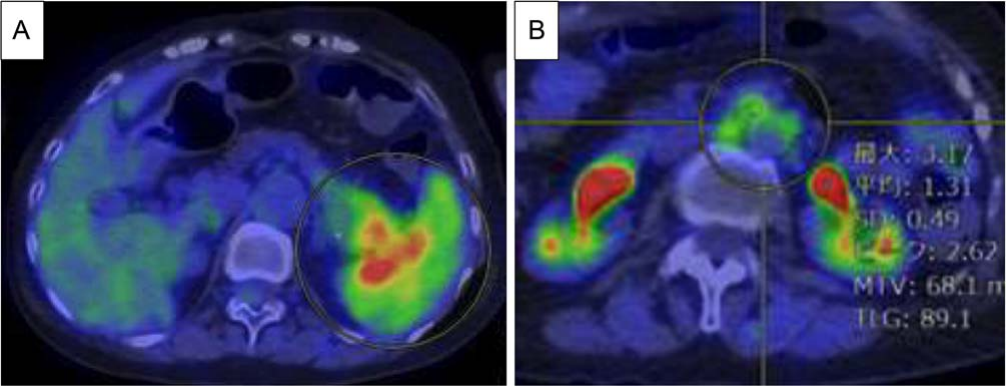
PET/CT before chemotherapy. (A) The lesion in the pancreatic tail exhibited high SUVmax value of 6.25 (yellow circle). (B) Para-aortic lymph nodes exhibited SUVmax value of 3.17 (yellow circle), and metastasis was suspected.

Endoscopic ultrasound-guided fine-needle aspiration (EUS-FNA) revealed atypical cell proliferation and PDAC was diagnosed. ^18^F-fluorodeoxyglucose (FDG) positron emission tomography (PET)/CT revealed increased FDG uptake in the pancreatic tail tumor (Fig. 2A), with a maximum standard uptake (SUVmax) of 6.25. Additionally, the para-aortic lymph nodes showed an SUVmax of 3.17 and metastasis was suspected (Fig. 2B). No liver or lung metastases were observed.

**Figure 3:**
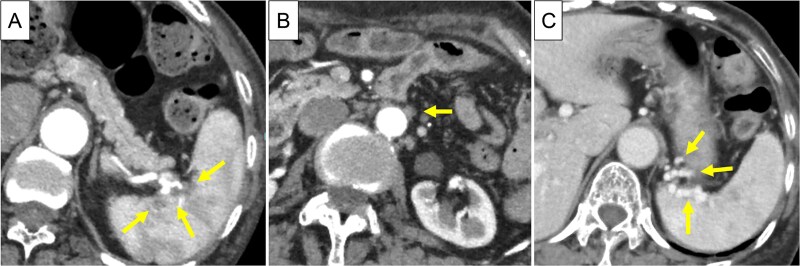
CE-CT after chemotherapy. (A) The tumor in pancreatic tail had shrunk to 3 cm in size (arrows). (B) The para-aortic lymph nodes were shrunk (arrow). (C) Collateral veins were amplified within the posterior wall of the upper gastric body (arrows).

**Figure 4:**
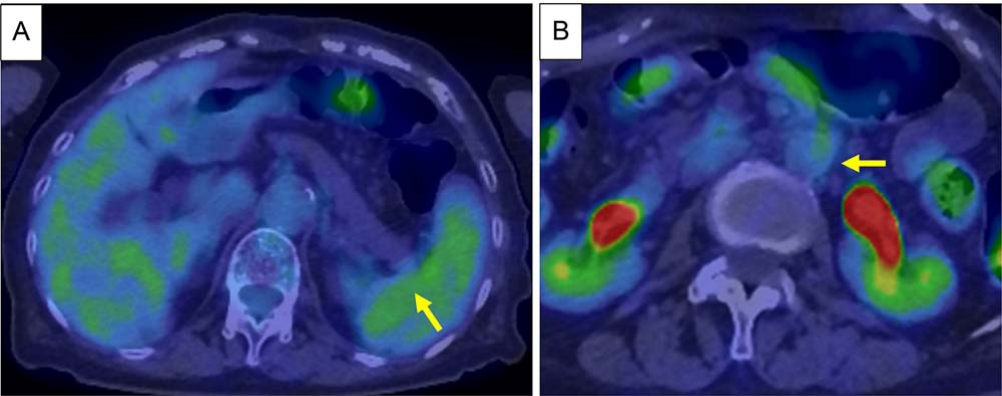
PET/CT after chemotherapy. (A) Decreased uptake was seen in the tumor in the pancreatic tail (arrow). (B) Decreased uptake was seen in the Para-aortic lymph nodes (arrow).

Based on these findings, the diagnosis was cT3N1M1 (LYM), cStage IV, according to the Union for International Cancer Control, 8th edition. Chemotherapy (gemcitabine + nanoparticle albumin-bound paclitaxel [GnP]) was initiated and nine courses were administered. CA19-9 decreased to 24.0 U/ml, CE-CT showed that the tumor had shrunk to 3 cm in size, and the para-aortic lymph nodes had shrunk (Fig. 3A and B). PET/CT revealed decreased uptake in the tumor and lymph nodes (Fig. 4A and B). The number of collateral veins increased further within the posterior wall of the gastric body (Fig. 3C).

**Figure 5:**
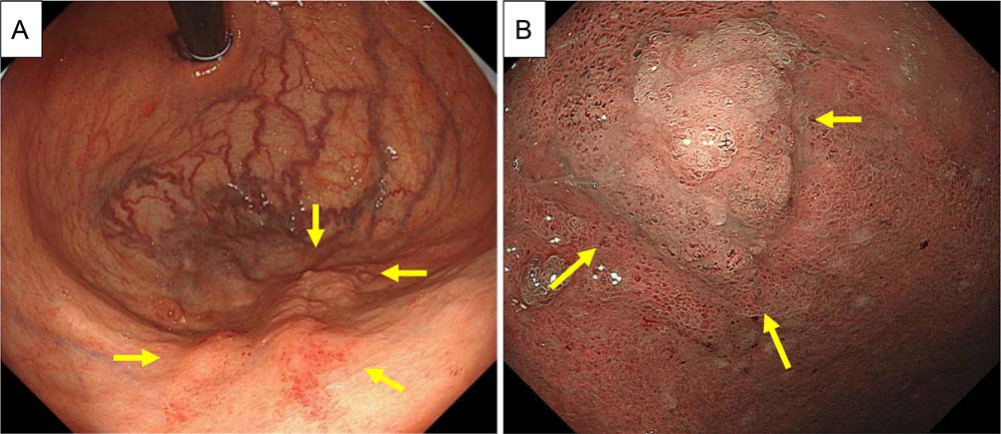
Preoperative EGD. (A and B) Preoperative EGD showed an irregularly shaped elevated lesion in the greater curvature of the gastric body (arrows), which was biopsied and found to be adenocarcinoma.

Therefore, the decision to perform surgical resection was made. Preoperative esophagogastroduodenoscopy revealed an irregularly shaped elevated lesion in the greater curvature of the gastric body (Fig. 5A and B), which was biopsied and found to be an adenocarcinoma. This site was not the puncture site found during EUS-FNA. Early-stage gastric cancer was considered.

**Figure 6:**
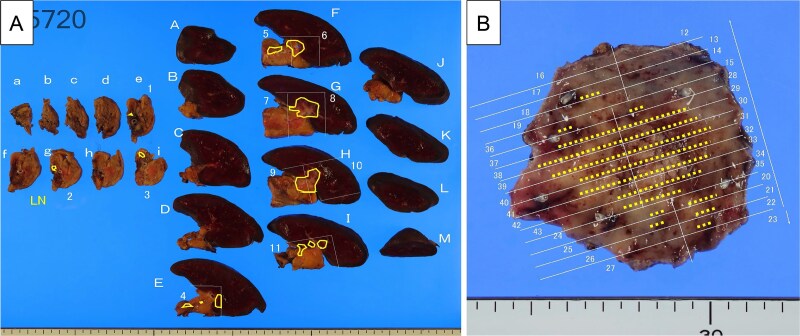
Resected specimens. (A) Pancreatic body, tail and spleen. The tumor indicated as invaded into the splenic artery and parenchyma (yellow line). (B) Resected gastric wall (40 × 35 mm). The yellow dots show the distribution of the adenocarcinoma (yellow dots).

**Figure 7:**
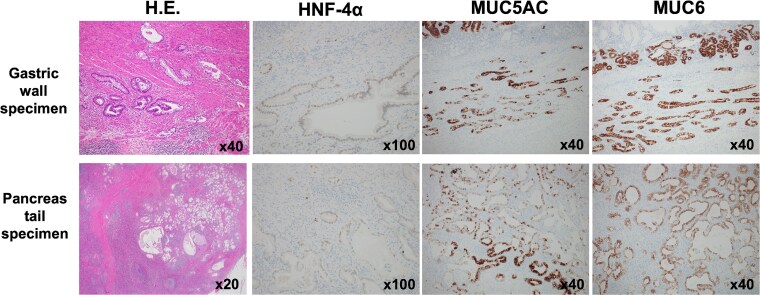
Immunohistochemistry. Immunohistochemistry of the gastric lesion revealed the same staining patterns as primary pancreatic cancer for HNF-4a, MUC5AC, and MUC6. Therefore, the pathological diagnosis was adenocarcinoma, consistent with metastasis of PDAC.

The pancreatic lesion, para-aortic lymph nodes, and gastric lesion were all considered resectable, and distal pancreatectomy, splenectomy, para-aortic lymph node dissection, and partial gastrectomy were performed (Fig. 6A and B). Pathological examination revealed a well-differentiated tubular adenocarcinoma in the submucosa and muscularis propria of the stomach wall. The tumor was not exposed to the serosa, and no lymph node involvement was observed around the stomach. Immunohistochemistry revealed the same staining patterns for HNF-4a, MUC5AC, and MUC6 as in primary pancreatic cancer (Fig. 7). Therefore, the pathological diagnosis was adenocarcinoma, consistent with metastasis of PDAC, rather than primary gastric cancer. The final pathological diagnosis of PDAC was pT3N1M1 (stomach, LYM), pStage IV.

A postoperative pancreatic fistula (ISGPS Grade B) was observed, and the patient was discharged 36 days after surgery. Nine months after surgery, lung metastasis was observed, and chemotherapy was restarted. Two years after the surgery, the patient was alive while undergoing chemotherapy for lung metastases; however, no recurrence was observed in the abdominal cavity.

## Discussion

The five pathways of secondary involvement of the gastrointestinal tract are as follows: (i) direct invasion, (ii) dissemination, (iii) lymphatic metastases, (iv) hematogenous metastases (v), and intraoperative seeding [3]. As the stomach is adjacent to the pancreas, direct invasion or peritoneal dissemination is common. However, in most patients with PDAC, although metastatic gastric tumors exist, patients may die before the examination to detect and confirm the presence of a gastric tumor.

Therefore, gastric metastases of PDAC are rare. Some metastases are hematogenous, whereas others develop in the puncture route via needle tract seeding (NTS) during transgastric EUS-FNA [4]. In this case, the site punctured using EUS-FNA was the posterior wall of the gastric body, whereas the gastric lesion was located on the greater curvature. Given the differing locations, NTS via puncture was considered unlikely, and the lesion was considered a hematogenous metastasis. Furthermore, the gastric tumor was localized in the submucosa and muscularis propria, was not exposed to the serosa, and there was no lymph node involvement around the stomach. Additionally, the developed collateral circulation in the gastric wall from the splenic vein might be the route of hematogenous metastasis caused by obstruction of the splenic vein in the splenic hilum by PDAC.

Yamada *et al*. summarized six cases of gastric metastasis from pancreatic cancer [5–10]. According to the report, five of six patients had a pancreatic tail or body origin, four presented metachronously, and four had metastases in other organs. Three patients underwent surgery for gastric metastasis, including two partial gastrectomies and one total gastrectomy. However, two patients died within 5 months. PDAC metastasis to the stomach constitutes stage IV disease; therefore, it is reasonable that the prognosis is poor.

Recently, the oncological advantages of surgery following neoadjuvant chemotherapy for patients with unresectable PDAC have been reported, and these treatments appear to significantly extend the survival period of patients with unresectable PDAC [11]. GnP is one of the standard regimens for advanced or metastatic PDAC, and several studies have indicated considerable benefits of neoadjuvant therapy with GnP for prolonged survival in PDAC. Improved tumor suppression contributes to patient selection and facilitates surgical procedures [12]. Therefore, not only surgical resection but also chemotherapy contributed to the prolonged prognosis in this case.

The rarity of gastric metastases from PDAC offers insights into its management and treatment. The patient’s clinical outcome underscores the necessity of an endoscopic workup of the stomach before surgery as a therapeutic approach.

## Data Availability

The data that support the findings of this study are available from the corresponding author upon reasonable request.
